# From algorithmic efficiency to cascading health burdens: a text-mining study of online food delivery riders in the platform economy

**DOI:** 10.3389/fpubh.2026.1868112

**Published:** 2026-07-22

**Authors:** Longxiao Li, Yongjun Zhou, Biyu Yang, Zhe Zhang

**Affiliations:** 1 School of Management, Chongqing University of Science and Technology, Chongqing, China; 2 School of Economics and Management, Hubei Minzu University, Enshi, China; 3 College of Mechanical and Vehicle Engineering, Chongqing University, Chongqing, China

**Keywords:** algorithmic management, mixed-methods research, occupational health burdens, online food delivery riders, topic modeling

## Abstract

Algorithmic management has markedly improved operational efficiency in the platform economy, yet its consequences for the occupational health of platform workers remain insufficiently understood. This study therefore examines how efficiency-driven algorithmic pressures produce occupational health burdens among online food delivery (OFD) riders in China. The analysis drew on 10,103 valid rider comments from a major Chinese social media platform, and it combined Latent Dirichlet Allocation topic modeling with co-occurrence network analysis and sentiment intensity assessment. Semi-structured interviews with 32 OFD riders were added to corroborate these computational findings and to reduce reliance on a single data source. Expert review identified five core health burden dimensions: physical exhaustion, social devaluation, disciplinary distress, injury vulnerability, and health-protection deficit. Network analysis showed that these dimensions form a tightly interconnected structure with physical exhaustion as the central hub, and that pressure cascades from operational demands into punitive mechanisms and then into injury risk and social protection deficits. A clear gap emerged between structural prominence and emotional intensity. Riders discussed time pressure most widely and largely accepted it as routine, while punitive mechanisms and health-protection deficits drew the strongest negative responses. The study contributes an integrated text-mining framework for occupational health research. It reframes rider health as a cascading system of mutually reinforcing burdens rather than a set of isolated risks, and it shows that the most central burden is not the one felt most acutely. These insights point to concrete measures for governments, platforms, rider organizations, and industry associations such as portable occupational-injury insurance, algorithmic transparency, fairer timing and rating rules, and accessible grievance channels.

## 1 Introduction

The rapid expansion of the digital platform economy has given rise to novel employment arrangements and reshaped global labor markets (1). At the heart of this transformation sits algorithmic management, which relies on big data and machine learning to handle resource allocation, task assignment, and performance evaluation in real time (2). The approach has been widely adopted in sectors such as ride-hailing and online food delivery (OFD), and has markedly improved operational efficiency (3). The OFD industry itself has grown into a major global sector. Tens of millions of workers are now engaged in this industry, and the sector generates a market value of more than 130 billion dollars (4). This growth reflects both the modernization of the catering sector and the evolution of urban service systems, and now forms a central part of the broader platform economy (5).

Algorithmic management has produced substantial economic gains as the platform economy continues to expand. However, the efficiency gains that drive platform growth also create systemic occupational health challenges for platform-dependent workers. OFD riders are particularly affected because their labor processes and income structures depend heavily on platform algorithms (6). Although algorithmic management has reduced transaction costs and offered more flexible earning opportunities (7), the deepening of algorithmic control has turned worker health protection into an urgent public health issue (8). The resulting health burden spans multiple dimensions and does not stay within a single domain. Consider first the physical toll. OFD riders often work extended hours under intense time pressure, and this pattern raises the risk of physical fatigue and musculoskeletal strain (8, 9). To meet these tight delivery deadlines, riders routinely run red lights and speed during deliveries (10). These behaviors raise the risk of traffic-related injuries and pose direct threats to physical safety (11, 12). The psychological toll runs in parallel. Income instability and occupational stigmatization contribute to persistent distress and produce anxiety and burnout (9). Underlying both sits a structural protection gap. The absence of adequate social insurance compounds these burdens and leaves riders without timely access to medical care or occupational injury compensation (5, 13, 14).

From a public health perspective, these challenges go far beyond ordinary labor disputes. Seen together, they form a cluster of occupational health burdens that now affect millions of workers in the expanding platform economy (8). The World Health Organization and the International Labour Organization have estimated that long working hours and occupational injuries cause hundreds of thousands of deaths each year (15). Exposure to long working hours alone accounts for approximately 745,000 deaths annually from ischemic heart disease and stroke, and represents the single largest occupational risk factor in the global burden of disease (16). OFD riders operate under algorithmic regimes that systematically compress rest time and transfer risk to individual workers. This makes them a particularly vulnerable occupational group, and the health outcomes of this group carry implications far beyond the workplace, reaching individual well-being, urban traffic safety, public healthcare expenditure, and social equity.

Research on the occupational health of platform riders has developed in stages. Early studies examined single problems, such as traffic injury (11), physical fatigue (9), or psychological distress, largely through interviews and case studies. A later body of work linked these problems to algorithmic management (9) and recognized that the burdens are multidimensional and psychosocial in nature (8). More recently, researchers have noted that data-driven analysis can complement these methods and reveal patterns of rider health at the population level (17, 18). The present study follows this trajectory and takes a further step, combining large-scale rider discourse with semi-structured interviews to examine how the different burdens connect and reinforce one another.

Within this development, two gaps remain. First, most studies examine the health dimensions in isolation and leave the structural links among them underexplored (17, 19). The Job Demands-Resources (JD-R) model holds that occupational stressors rarely operate independently and tend to interact and produce compounding effects (20), yet few empirical studies have mapped how algorithmic pressures connect and cascade across multiple health burdens among OFD riders. Second, the evidence still rests mainly on case studies, interviews, and surveys. These methods capture individual accounts well but are limited in representing the collective health experience and emotional states of the broader rider population (17, 18). Large-scale text data analyzed with data-driven methods offer a complementary route to population-level patterns.

Motivated by these considerations, the present study conducts a systematic analysis of the multi-dimensional occupational health burdens faced by OFD riders under algorithmic management, and the analysis proceeds in three steps. First, Latent Dirichlet Allocation (LDA) topic modeling identifies and categorizes core health burden topics from unstructured text data. Second, sentiment intensity analysis quantifies the emotional weight of each extracted topic and exposes the gap between the objective distribution of different health burdens and the subjective psychological impact they carry for OFD riders. Third, a topic co-occurrence network is constructed, and structural centrality metrics are then applied to identify critical hub nodes and trace the transmission pathways through which algorithmic pressures cascade across health burden dimensions.

This study offers three main contributions. Theoretically, it extends the JD-R model from the interaction of separate demands and resources toward the structural transmission of occupational health burdens. Topic co-occurrence network analysis shows that the burdens faced by OFD riders form an interconnected and cascading system, and reveals that the burden at the center of this system differs from the burden that riders feel most acutely. Methodologically, the study develops a mixed-methods framework for occupational health research in the platform economy. It applies LDA topic modeling, co-occurrence network analysis, and sentiment intensity analysis to large-scale rider discourse, and it adds semi-structured interviews with OFD riders. The computational strand captures health experiences at the population level, while the interviews corroborate the five health burden dimensions and ground them in concrete rider experience, so that the two strands combine breadth and depth. Practically, the findings provide concrete guidance for governments, platforms, rider organizations, and industry associations, including portable occupational-injury insurance, algorithmic transparency, fairer timing and rating rules, and accessible grievance channels. These contributions advance understanding of how algorithmic management reshapes occupational health in the platform economy, and they provide evidence for targeted public health interventions.

The remainder of this paper is organized as follows. Section 2 reviews the literature on algorithmic management and occupational health. Section 3 describes the research methodology. Section 4 presents the data analysis results. Section 5 discusses the influence of algorithmic management on occupational health and the systemic connections among the identified health burden dimensions. Section 6 concludes the study.

## 2 Literature review

### 2.1 Algorithmic management and occupational health burdens

Algorithmic management is a defining feature of digital platforms. It covers the use of algorithmic systems for real-time monitoring, task allocation, performance evaluation, and reward-punishment decisions directed at platform workers (21, 22). Its impact on worker health and safety has drawn substantial academic attention (8, 11, 14). Although algorithmic management optimizes logistical paths and improves operational transparency, its efficiency-first logic and opaque mechanisms also generate systemic occupational health burdens (17, 22).

The health effects of algorithmic control generally appear across three dimensions. First, the continuous pursuit of efficiency through persistent monitoring drives workers into high-intensity labor and often causes physical fatigue, musculoskeletal disorders, and psychological burnout (8, 23). In the OFD sector, compressed delivery windows push riders into risky behaviors that directly compromise traffic safety and raise the probability of occupational injuries. Second, piece-rate compensation and dynamic pricing mechanisms create income volatility that amplifies financial stress and psychological distress (7, 10). Research has established that piece-rate pay links to worse health outcomes than salaried employment, since it encourages workers to forgo rest and push beyond safe physical limits (24). Algorithmic reputation systems and rating-based control further amplify psychological strain by tying worker identity to platform-assigned scores (25). Third, platforms classify workers as independent partners. This classification allows platforms to circumvent traditional employer liabilities and worsens the dual burden of low wages and intensive labor (26). This regulatory ambiguity leaves workers without fundamental social protections such as occupational injury insurance and medical coverage (14). Even where regional pilot programs for occupational injury insurance operate, coverage remains fragmented and inadequate (27, 28). The structural imbalance between technological rationality and health protection has thus become a widespread public health challenge across the global platform economy (17, 29). These three lines of research clarify how physical, financial, and institutional pressures each weigh on rider health. They also leave one question open. Each line tends to treat its own burden on its own terms, so how these pressures connect and reinforce one another in a single rider’s working day has received less attention.

A growing body of public health research has begun to quantify these effects. Studies on Chinese OFD riders have reported moderate-to-high burnout prevalence. Recent evidence from the same setting further indicates that features of algorithmic management are closely associated with the level of rider burnout (8). Organizational factors such as ranking systems and punishment mechanisms significantly predict burnout levels (8). Labor intensity among delivery riders has been linked to reduced physical and mental health outcomes through mediation pathways involving fatigue and overwork (30). Across these studies, heavier algorithmic demands tend to accompany poorer rider health. The studies establish this association well, but they say less about the process that links the two. The JD-R model speaks to this process. It holds that excessive job demands deplete workers’ physical and psychological resources and lead to health impairment, while insufficient job resources reduce the capacity to buffer against these demands (20). This study draws on the model to examine how the demands and resource deficits of algorithmic management connect and cascade across the health burden dimensions.

### 2.2 Toward a multidimensional analysis of occupational health burdens

Research on the occupational health effects of algorithmic management has built a solid empirical base (31). Much of this work relies on in-depth interviews, participant observation, and localized case studies (2, 17), and it shows how algorithmic management consolidates platform authority and reshapes the daily experience of workers (32). These qualitative approaches have uncovered the micro-mechanisms of algorithmic control, including opaque decision-making, rigorous performance metrics, and entrenched power asymmetries (7, 33, 34). These studies offer rich, context-sensitive insight into how such control operates in practice. Because their strength lies in rich individual detail, large-scale and data-driven methods can complement them by showing how widely each health burden is distributed across the workforce.

Alongside this methodological work, empirical studies have mapped specific facets of rider health. The evidence now covers burnout and psychological strain (8, 35), occupational injury and traffic safety (12, 26), and healthcare access deficits (14). More recent studies have begun to connect these facets and to call for a more integrated view (18, 36). What remains underdeveloped is a systematic account of how the burdens connect and reinforce one another within the same group of riders. This question matters because the occupational health literature has long held that workplace hazards rarely act in isolation, and that physical, psychological, and organizational risk factors interact to produce cascading health outcomes (36). An analytical framework that captures these interactions can give a more complete assessment of the health burden that algorithmic management imposes, and it can guide targeted occupational health interventions. The present study sets out to build such an account.

Despite substantial theoretical groundwork, building a systematic framework to map the occupational health burden profile of algorithmic management remains a vital research area. To evaluate these complex dynamics, the study combines topic modeling, sentiment analysis, and semantic network analysis into a unified methodological framework. An LDA model, validated by coherence and perplexity metrics, first extracts core health burden topics and quantifies their sentiment intensities. Topological indicators such as degree centrality and betweenness centrality are then applied to the topic co-occurrence network. These indicators uncover the underlying structural mechanisms and transmission pathways through which algorithmic pressures cascade across health burden dimensions.

## 3 Materials and methods

To identify the core dimensions of occupational health burdens under algorithmic management and to investigate their underlying structural mechanisms, this study integrates LDA topic modeling with structural network topology and sentiment intensity analyses.

### 3.1 Latent Dirichlet allocation

The proliferation of digital platforms has generated a vast accumulation of unstructured text data. Traditional text processing techniques rely mainly on predefined rules or manual intervention. These techniques often encounter scalability limitations and subjective human bias when applied to large-scale datasets (37). Topic modeling has therefore emerged as an effective unsupervised text mining approach for uncovering latent semantic structures based on word co-occurrence patterns. The primary advantage of this approach is its data-driven objectivity, which provides an impartial framework for analyzing complex corpora without requiring prior manual annotation (3, 38).

Among topic modeling techniques, LDA remains a foundational approach because of its broad applicability and reliable performance (39). LDA uses a hierarchical document-topic-word probabilistic structure and infers latent topics directly from explicit textual features. This generative process captures intricate semantic relationships and effectively mitigates the subjectivity inherent in traditional manual coding (37, 40). Beyond these general strengths, two features of LDA are particularly important for the present study. First, because LDA represents each topic as an explicit and interpretable distribution over words and each document as a distribution over topics, the resulting themes can be read directly from their characteristic words and validated by domain experts while avoiding the opacity of dense latent vectors (41), and through its Dirichlet priors the model offers principled control over the sparsity and smoothness of these distributions. Second, this combination of interpretability and reliability has made LDA one of the most widely adopted topic models for short social media text in very recent research on public health and platform labor, where it is routinely paired with sentiment analysis and with co-occurrence or semantic network analysis of the extracted themes, much as in the present study (38, 42–44). It has, moreover, been applied directly to food delivery platform workers and the broader gig economy under algorithmic management (18, 40), a setting closely aligned with this study.

#### 3.1.1 Mathematical formulation of LDA

**Figure 1:**
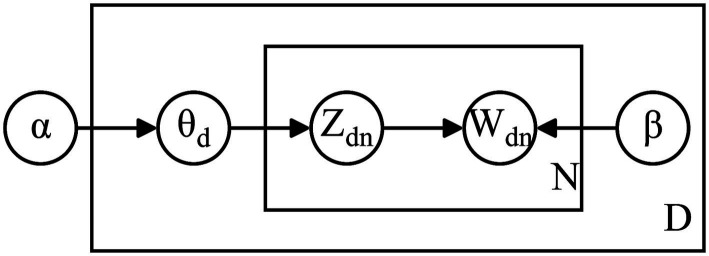
Graphical model of LDA.

The generative process of LDA begins with each topic drawing a word distribution from a Dirichlet prior parameterized by β. Concurrently, each document d draws a topic distribution θ_d_ from a Dirichlet prior parameterized by α. To generate the n -th word $W_{\mathrm{dn}}$ within document d , a specific topic assignment $Z_{\mathrm{dn}}$ is initially sampled from the document-level topic distribution θ_d_. The observed word $W_{\mathrm{dn}}$ is subsequently drawn from the word distribution corresponding to the assigned topic $Z_{\mathrm{dn}}$ . Within the graphical model framework, plate notation D defines the total number of documents in the corpus, and N represents the total number of word tokens within document d . The generative logic of the LDA model is illustrated in Figure 1. The notation characterizing the LDA model is defined in Table 1.

**Table 1:** Notation of the LDA model.

Core element	Symbol	Description
Structural parameter	α	Hyperparameter driving the generation of document-level topic proportion distribution
	β	Hyperparameter driving the generation of topic-level word probability distribution
Latent dimension	$\theta_{d}$	Topic probability distribution of document d
	$Z_{\mathrm{dn}}$	Topic assignment variable for the n -*th* word in document d
Observed feature	$W_{\mathrm{dn}}$	The n -th observed word in document d
Structural parameter	N	The total number of word tokens within document d
	D	The total number of documents in the collection

#### 3.1.2 Model evaluation metrics

To validate the quality and interpretability of the extracted topics, perplexity and coherence scores are adopted as the primary evaluation metrics (45).

Perplexity quantifies the predictive capacity of the LDA model on unseen data. It is calculated as the exponential of the negative log-likelihood of the test corpus normalized by the total word count. Lower perplexity values indicate higher accuracy in predicting word distributions within unseen documents (41). The mathematical expression is formulated as Equation 1.

$$\text{perplexity}(D)=\exp\{-\frac{\sum_{d=1}^{|D|}\text{logL}(w_{d}|\theta_{d})}{\sum_{d=1}^{|D|}N_{d}}\}$$ (1)

In this equation, D represents the document corpus, $N_{d}$ indicates the number of terms within document d , and $L(w_{d}|\theta_{d})$ is the likelihood function for document d .

Coherence serves as a complementary metric that assesses the semantic correlation among word tokens within a topic. It counterbalances the tendency of perplexity to occasionally favor overfitted models. Higher scores signify stronger logical consistency within the extracted topics and support robust academic interpretation. To determine the semantic consistency among the top M words of topic k, the coherence metric is calculated using Equation 2.

$$c_{\text{UMass}}^{\left(k\right)}=\sum_{m=2}^{M}\sum_{l=1}^{m-1}\log\frac{D(w_{m},w_{l})+\epsilon }{D(w_{l})}$$ (2)

In this formulation, $D(w_{m},w_{l})$ denotes the document count containing both $w_{m}$ and $w_{l}$ , whereas $D(w_{l})$ signifies the document count containing $w_{l}$ alone. A small smoothing constant ϵ is incorporated to prevent division by zero and ensure numerical stability.

### 3.2 Structural topology and centrality models

#### 3.2.1 Mathematical formulation of the co-occurrence network

To map the structural interactions among the identified occupational health burden dimensions, this study constructs a topic co-occurrence network. Within this network, nodes represent the extracted health burden topics, and edges represent their co-occurrence within individual comments. Assigning attribute weights to each node requires calculating the overall sentiment intensity for each specific health burden topic.

Drawing upon feature-based sentiment computation methods (46), a weighted average approach is employed to aggregate document-level sentiment scores into topic-level metrics. Let $R=\{r_{1},r_{2},\ldots ,r_{n}\}$ denote the set of OFD rider comments, and $T=\{t,t_{2},\ldots ,t_{m}\}$ represent the set of dilemma topics extracted by LDA. The output matrix generated by LDA model is defined as $\;W_{n\times m}$ , where the element $w_{i,j}\;$ indicates the probability weight of topic $t_{j}$ within comment $r_{i}$ . For each specific topic $t_{j}$ , the aggregated sentiment score $t_{s_{j}}$ is calculated using Equation 3.

$$t_{s_{j}}=\frac{\sum_{i=1}^{n}w_{i,j}\cdot s_{i}}{\sum_{i=1}^{n}w_{i,j}}$$ (3)

In this equation, $t_{s_{j}}$ represents the overall sentiment score of the j -th dilemma topic, n is the total number of comments in the corpus, $w_{i,j}$ denotes the weight distribution of the i -th comment on the j -th topic, and $s_{i}$ is the specific sentiment score of the i -th comment.

#### 3.2.2 Centrality metrics

The structural characteristics of the co-occurrence network are evaluated using two primary centrality metrics.

Degree centrality measures the extent of a node’s direct connections with other nodes. A topic with high degree centrality is more likely to co-occur with other health burden dimensions. Raw centrality values increase with the number of nodes in a network, so degree centrality and betweenness centrality were both normalized. Without normalization, these values would mainly reflect network size and obscure a node’s relative position within the structure. Following Freeman’s formalization, degree centrality was divided by the largest degree a node can reach in a network of n nodes, which equals n−1 . Betweenness centrality was divided by its theoretical maximum for a network of the same size. As a result, both measures lie within a common range from 0 to 1, and this shared scale makes the centrality of keywords directly comparable across the topic dimensions. Equation 4 expresses the standardized degree centrality.

$$C_{D}(p_{k})=\frac{\sum_{i=1}^{n}a(p_{i},p_{k})}{n-1}$$ (4)

In this formulation, $p_{k}$ represents the relative degree centrality of the target node, n represents the total number of nodes in the network, and $a(p_{i},p_{k})$ is an element of the adjacency matrix where a value of 1 indicates a connection between node $p_{i}$ and $p_{k}$ , with 0 indicating otherwise. The n−1 represents the theoretical maximum number of possible connections, incorporated specifically for normalization.

Betweenness centrality measures the extent to which a node acts as a bridge along the shortest paths between other node pairs. Topics exhibiting high betweenness centrality operate as critical structural intersections within the network. The probability distribution of a target node $p_{k}\;$ falling on the shortest path between nodes $p_{i}$ and $p_{j}$ is calculated using Equation 5.

$$b_{\mathrm{ij}}(p_{k})=\frac{g_{\mathrm{ij}}(p_{k})}{g_{\mathrm{ij}}}$$ (5)

In this equation, $g_{\mathrm{ij}}$ represents the total number of shortest paths between node $p_{i}\;$ and node $p_{j}$ , whereas $g_{\mathrm{ij}}(p_{k})\;$ represents the number of those shortest paths passing through node $p_{k}$ . Consequently, the overall betweenness centrality index $C_{B}(p_{k})$ for node $p_{k}\;$ is the sum of the probability distributions for all node pairs in the network, as presented in Equation 6. For cross-topic comparison, betweenness centrality values were subsequently normalized to the [0, 1] interval using Equation 7.

$$C_{B}(p_{k})=\sum_{i=1}^{n}\sum_{j<i}^{n}b_{\mathrm{ij}}(p_{k})$$ (6)

The min-max normalization in Equation 7 maps these centrality values to a standard interval of [0,1] . For a given set of original scores $\left\{s_{k}\right\}$ , the normalized score $\tilde{s_{k}}$ is computed, where min and max denote the minimum and maximum values of the original score set, respectively.

$$\tilde{s_{k}}=\frac{s_{k}-\min}{\max-\min}$$ (7)

### 3.3 Inferential framework for health burden identification

A methodological clarification is warranted regarding the inferential relationship between text-derived topics and occupational health burdens. The LDA model identifies latent semantic clusters based on word co-occurrence patterns within rider-generated text. These clusters reflect the thematic structure of rider discourse. They are not clinically measured health outcomes. The health burden labels assigned to each topic are therefore analytical interpretations grounded in the semantic content of high-probability keywords and validated through expert review. They are not direct measurements of physiological or mental health status.

This distinction carries important implications for the interpretation of the findings. When this study reports that physical exhaustion occupies a central position in the co-occurrence network, it means that discourse about time pressure is structurally central in how riders articulate their occupational concerns. The inference that such discourse reflects genuine physical strain rests on two supporting grounds. First, the keyword composition of this topic includes terms such as overtime, delivery time, and food preparation. These terms align closely with the job demand constructs that the occupational health literature has empirically linked to fatigue, musculoskeletal disorders, and burnout (8, 20, 30). Second, the expert panel, composed of 25 experienced riders and domain specialists, confirmed that these textual patterns correspond to lived occupational health experiences. The same logic applies to the sentiment intensity scores. A low sentiment score for a given topic indicates that rider discourse about that topic carries a predominantly negative affective tone. This finding is consistent with, but not identical to, elevated psychological distress at the individual level.

This text-as-evidence approach complements clinical and survey-based health assessments and does not replace them. Its value lies in capturing population-level patterns of health-related concern at a scale and ecological validity that structured instruments cannot easily achieve. The limitations of this inferential step are further discussed in Section 6.3.

### 3.4 Data collection and processing

#### 3.4.1 Online comment data

The data for this study come from Douyin, a major short-video platform in China on which many OFD riders publicly discuss their work experiences and health concerns. To construct the research corpus, a web crawler was deployed in March 2025 to collect 10,832 user comments. The search strategy incorporated specific keywords, including rider, knight, and OFD delivery rider. Throughout the data acquisition process, strict ethical standards were maintained. All personally identifiable information, including user IDs, nicknames, and geolocation data, was completely anonymized to protect user privacy. The study analyzed publicly available social media comments. No personally identifiable information was collected or stored. The study did not involve direct human participation, clinical interventions, or biological samples, and therefore did not require institutional ethics committee approval.

To ensure data quality and analytical validity, the 10,832 raw comments underwent an initial filtering process. Duplicate comments, automated platform replies, and excessively short texts lacking substantive meaning were excluded. This process resulted in a baseline dataset of 10,103 valid comments. A three-stage preprocessing procedure was subsequently implemented to prepare this dataset for LDA topic modeling and network analysis.

The first stage involved text cleaning and noise reduction (37). Regular expressions were applied to filter out non-textual noise, including emoticons, URLs, and non-Chinese characters. Stop words were then eliminated using the combined HIT and Baidu Chinese stop-word lists (47), supplemented by 187 domain-specific terms related to platform activities and new user benefits. In the second stage, core vocabulary was extracted through word segmentation with the Jieba segmenter (48) and part-of-speech tagging. Retaining only content words, namely nouns, verbs, and adjectives, yielded a focused lexicon of 523 terms. This vocabulary comprehensively covers key concepts identified in the corpus, such as overtime, running a red light, and insurance. The third stage focused on text structuring and vectorization. Using a Bag-of-Words approach, the textual data were converted into a numerical format. Term frequencies within each comment were calculated to generate a document-term matrix with a sparsity of 0.82. This matrix served as the structured input for the subsequent LDA model.

**Figure 2:**
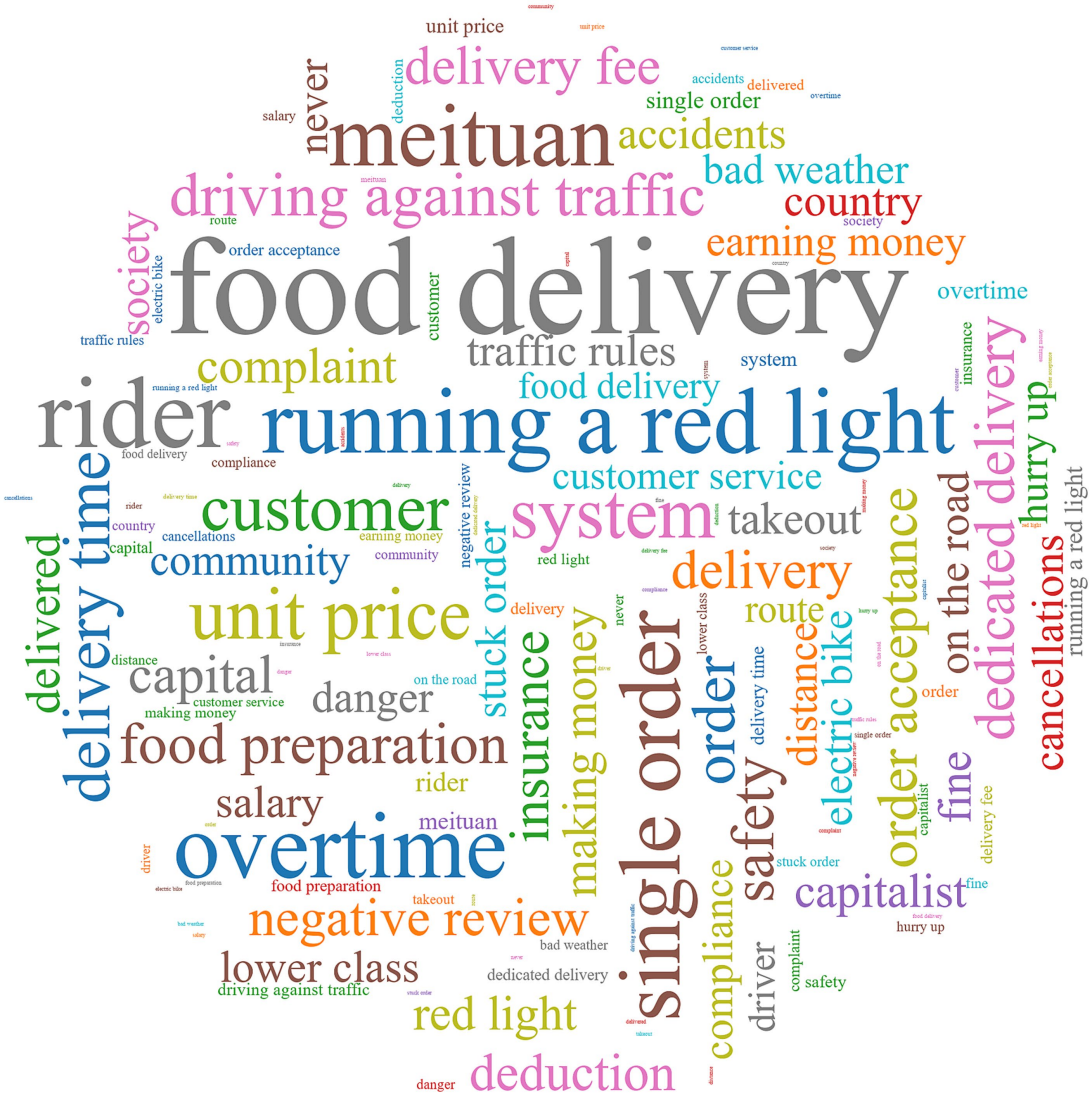
Word cloud of OFD rider comments.

Following data cleaning and tokenization, a word cloud was generated to visualize the term frequency distribution across the processed corpus. As shown in Figure 2, high-frequency terms such as overtime, running a red light, and fine feature prominently in OFD rider discourse. These recurring terms reflect the primary health-related concerns of riders under algorithmic management. They also provide a preliminary overview of the occupational health challenges that the subsequent topic modeling analysis examines in greater depth.

#### 3.4.2 Semi-structured interviews

To corroborate the computational findings and to partly offset the reliance of the text-mining analysis on a single social media platform, a supplementary interview study was conducted. The interviews were designed to examine the five health-burden dimensions derived from the text corpus against riders’ first-hand accounts, thereby moving from platform discourse to lived experience. They were intended to corroborate an existing dimensional framework, and were therefore deliberately focused and structured around the five dimensions.

Between April and May 2026, 32 OFD riders were recruited near commercial districts in Chongqing, a mountainous municipality in southwestern China whose steep terrain makes the physical demands of delivery work especially salient. Participants were purposively sampled to span different platforms and employment arrangements. The sample comprised 24 male and 8 female riders aged between 22 and 44 years, with educational attainment ranging from primary school to a bachelor’s degree. Most participants worked for Meituan, with the remainder distributed across Taobao Shangou (formerly Ele.me), and JD Miaosong. The sample was evenly divided between station-based dedicated riders and crowdsourced riders, with platform tenure ranging from 6 months to 5 years. Detailed participant characteristics are reported in Table 2.

**Table 2:** Profile of interview participants (*N* = 32).

Variable	Category	n
Gender	Male	24
	Female	8
Age (years)	22–29	12
	30–39	15
	40–44	5
Education	Primary school	2
	Junior high school	8
	Senior high school	11
	Secondary vocational	3
	Junior college	7
	Bachelor’s degree	1
Platform	Meituan	25
	Taobao Shangou (formerly Ele.me)	5
	JD Miaosong	2
Employment type	Dedicated	16
	Crowdsourced	16
Tenure (years)	≤1	7
	>1 to 3	17
	>3	8

To ensure thematic alignment with the computational analysis, the interview guide was organized around five open-ended questions, with each question corresponding to one of the five health-burden dimensions identified through topic modeling. The interview protocol was reviewed and approved by the Ethics Committee of Chongqing University of Science and Technology (approval no. 2026LLSC0012). All participants were informed of the study’s purpose, the voluntary nature of participation, and their right to withdraw at any time, and all provided written informed consent before being interviewed; every interview was conducted and all records were stored under strict anonymity to protect participant privacy.

Interview recordings were transcribed and analyzed thematically using the framework method (49), in which all 32 transcripts were coded deductively against the five health-burden dimensions. Representative excerpts were translated into English by the authors for presentation. The accounts of injuries and accidents reported below are based on participants’ own reports rather than official accident registries, because riders engaged under flexible-employment partnership arrangements are generally not covered by the statutory work injury insurance system.

## 4 Results

### 4.1 Identification of latent topics

#### 4.1.1 Determination of the optimal number of topics

Determining the optimal number of topics is essential for ensuring model performance and interpretability. To balance these considerations, this study adopted a composite indicator combining perplexity and topic coherence. The indicator was used to balance statistical fit with semantic clarity. To minimize random error, the model was trained iteratively across a topic range of 1 to 9, with 10 independent runs per setting.

Semantic interpretability was initially evaluated through the coherence score, which measures the degree of semantic similarity among high-scoring words within a given topic. As the number of topics approached 5, the coherence score reached its peak.

To further validate the statistical robustness of the model, perplexity was evaluated across the same range of topic numbers. As a metric of predictive performance, perplexity quantifies the alignment between the probability distribution predicted by the model and the actual word distribution within the corpus. The results indicate that the model attained its minimum perplexity score at the five-topic threshold, which demonstrates optimal predictive performance.

**Figure 3:**
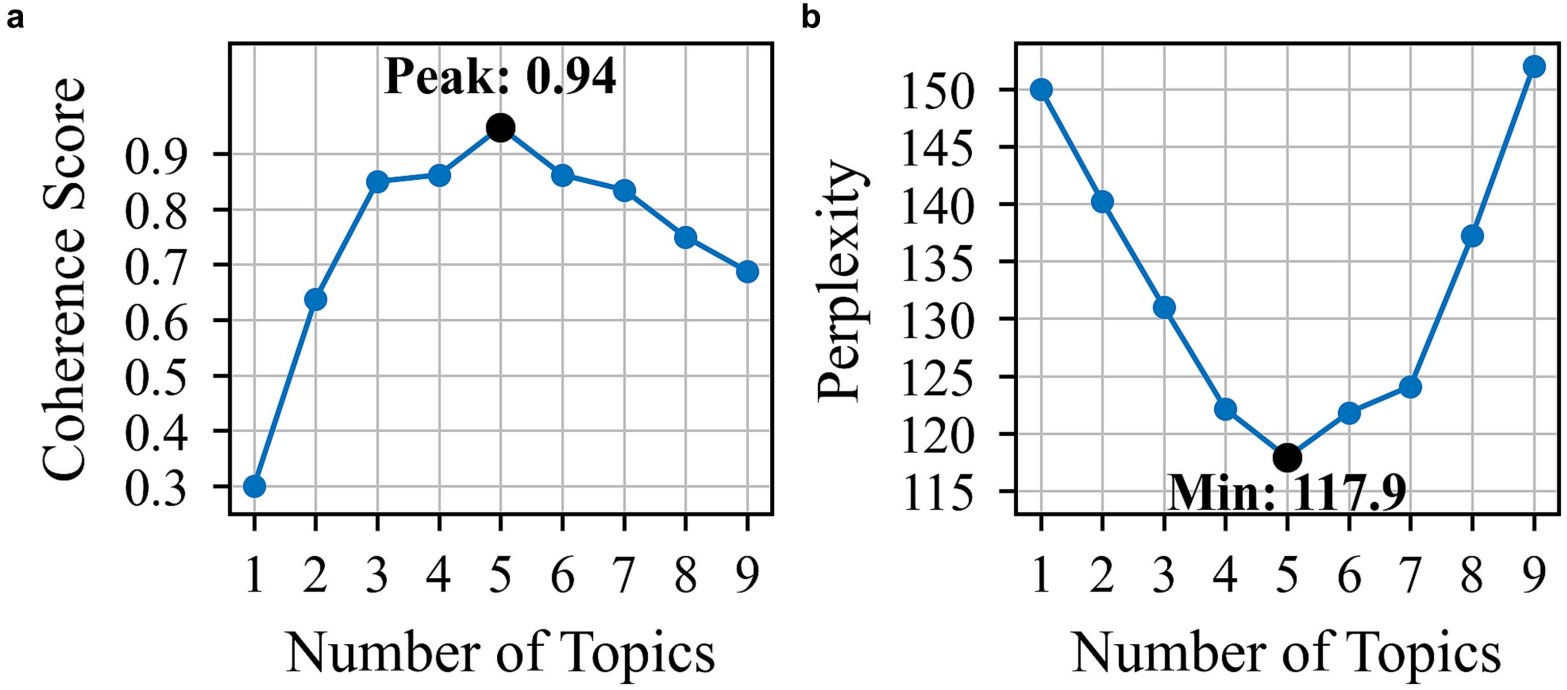
Topic coherence and perplexity across different numbers of topics. **(a)** Semantic Coherence. **(b)** Predictive Perplexity.

Given the convergence of peak semantic coherence and minimum perplexity illustrated in Figure 3, the optimal number of topics was determined as five for all subsequent analyses. This dual-indicator evaluation ensures that the derived topic structure is both mathematically robust and suitable for examining the multi-dimensional occupational health burdens among OFD riders.

To verify the robustness of the selected topic number, we conducted two complementary checks, using the preprocessing pipeline of Section 3.4.1. The document-term dictionary was constructed in Gensim (50) and filtered with three parameters, namely no below set to 2, no above set to 0.7, and keep n set to 1,000. All models were trained over 20 passes and 200 iterations.

**Figure 4:**
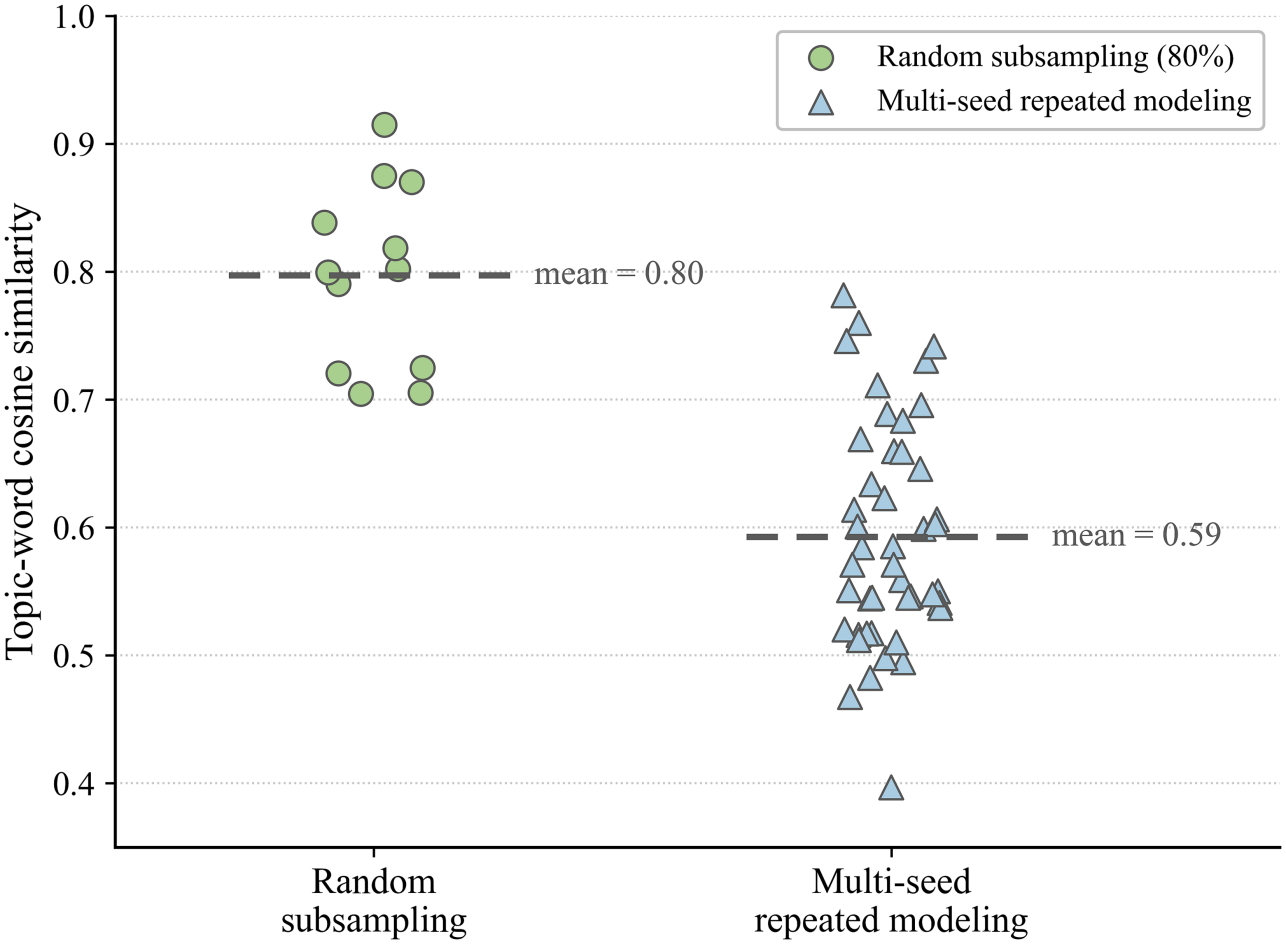
Topic stability across random seeds and subsamples.

The first examined stability under random initialization and data perturbation. Holding the topic number at five, we retrained the model across 10 random seeds and on 12 random subsamples each drawn at 80% of the corpus, aligned every solution to the baseline by optimal topic matching, and measured the cosine similarity of the topic and word distributions (51). As shown in Figure 4, mean similarity was 0.80 under subsampling and 0.59 across seeds, the lower seed value reflecting a known property of LDA on short, lexically overlapping comment text. The five-topic solution therefore remained robust.

**Figure 5:**
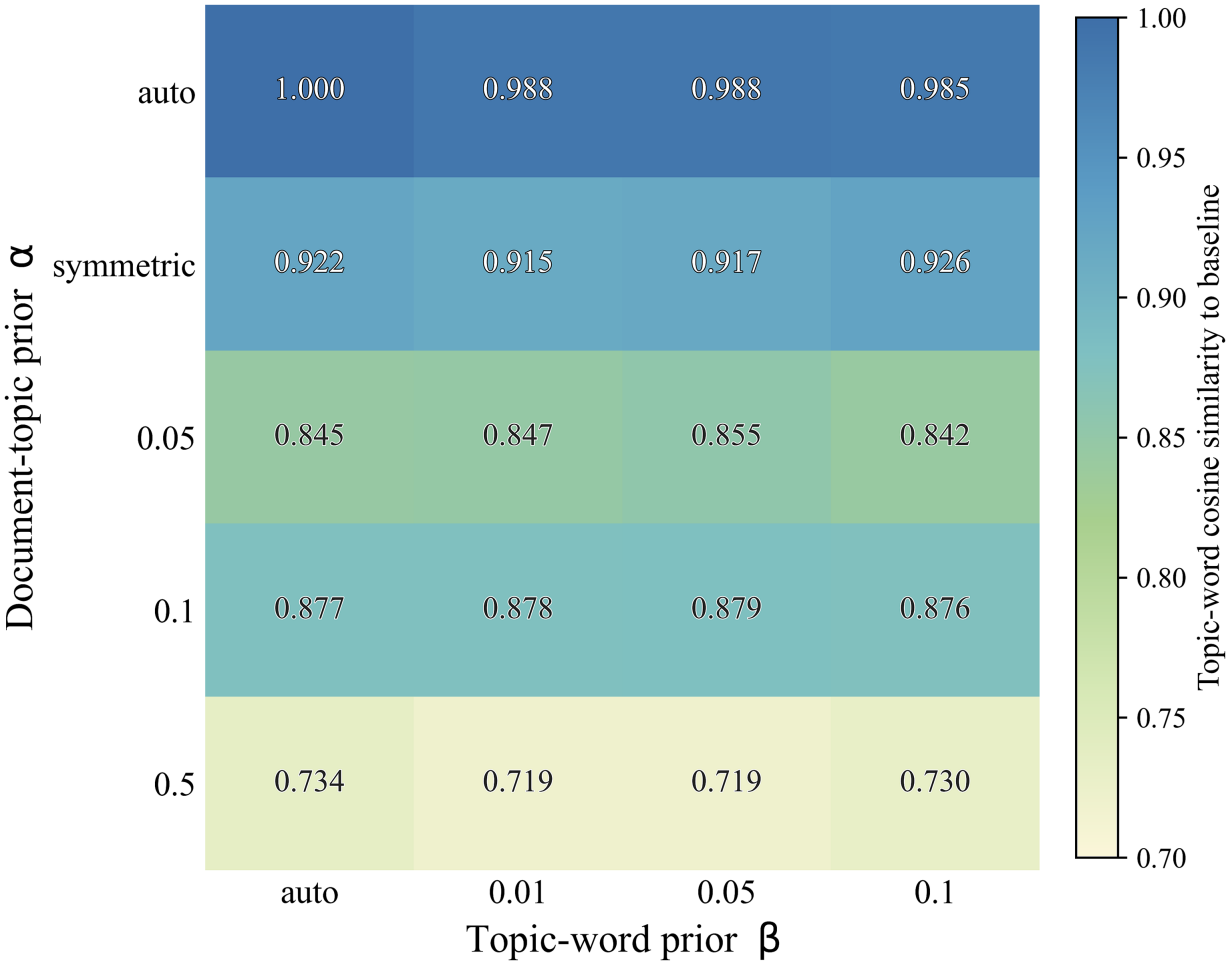
Topic stability across hyperparameters.

The second examined stability under hyperparameter variation. Holding the topic number at five, we retrained the model across a grid of Dirichlet priors that combined five document-topic priors (auto, symmetric, 0.05, 0.1, and 0.5) with four topic-word priors (auto, 0.01, 0.05, and 0.1). As Figure 5 shows, the cosine similarity to the baseline stayed above 0.84 across most of the grid and above 0.71 even under the most extreme prior of 0.5, and all five health-burden dimensions remained clearly identifiable through their characteristic keywords, with only the ordering of less frequent terms shifting. Together with the coherence and perplexity results reported above, these checks confirm that the five-topic structure is a stable and interpretable partition of the corpus.

#### 4.1.2 Hyperparameter configuration

Following the determination of K=5 , the Dirichlet hyperparameters α and β were subsequently configured. The results indicated that topic coherence optimized at α ≈ 10.0, derived from 50/K , and β = 0.01. This configuration aligns with the linguistic characteristics of the corpus. A higher α value promotes a more balanced topic distribution within individual comments. This setting reflects the tendency of OFD riders to discuss multiple intersecting issues in a single comment. A low β value promotes sparsity within the word-topic distributions and ensures that primary keywords, such as overtime and running a red light, maintain distinctively high probability weights. Consequently, this parameter differentiation sharpens topic boundaries and enhances overall semantic interpretability. To ensure robust model convergence and prevent underfitting, the training process was executed over 1,000 iterations.

#### 4.1.3 Initial topic generation

Based on the finalized hyperparameters, the LDA model was implemented to extract five distinct topics, each characterized by its 10 most probable keywords. These extracted topics provide a foundational mapping of the multi-dimensional occupational health burdens experienced by OFD riders. Table 3 summarizes the inferred topic labels alongside their corresponding keyword probability distributions.

**Table 3:** Dilemma topics and top 10 high-frequency words.

Topic no.	Topic label	Top 10 high-frequency words (with weights)
1	Physical exhaustion	Rider (0.108), order (0.065), delivery time (0.035), order acceptance (0.025), customer (0.025), single order (0.024), food preparation (0.021), delivery fee (0.020), order (0.019), Meituan (0.018)
2	Social devaluation	Takeout (0.219), Meituan (0.060), exploitation (0.035), traffic rules (0.016), country (0.016), lower class (0.016), society (0.015), compliance (0.015), danger (0.014), capitalist (0.013)
3	Disciplinary distress	Overtime (0.107), takeout (0.086), system (0.054), negative review (0.024), complaint (0.020), deduction (0.020), delivered (0.019), fine (0.017), delivery (0.015), customer (0.014)
4	Injury vulnerability	Running a red light (0.134), food delivery (0.085), driving against traffic (0.042), safety (0.042), unit price (0.033), red light (0.023), electric bike (0.015), ride-hailing (0.013), salary (0.012), driver (0.012)
5	Health-protection deficit	Food delivery (0.060), compression (0.049), insurance (0.038), capital (0.036) delivery riders (0.025), cancelations (0.022), industry (0.021), Meituan (0.020), accidents (0.020), dedicated delivery (0.020)

#### 4.1.4 Validation and structural integration of health burden dimensions

To ensure the semantic clarity and empirical validity of the initial topics, a tripartite expert panel was convened following established procedures in topic model validation (52). This panel comprised 25 senior OFD riders with more than 3 years of field experience, three platform operations specialists proficient in dispatch and pricing algorithms, and two researchers specializing in platform labor and occupational health studies. The panel was recruited between July 15 and August 20, 2025.

During the validation process, the panel first evaluated the granularity of the topic extraction. All panel members confirmed that the five derived categories accurately represented the occupational health challenges of the OFD labor process. The categorization was considered neither redundant nor excessively fragmented. The five initial topics were then synthesized and aligned with the multi-dimensional framework of occupational health burdens grounded in established theoretical foundations and workflow correlations. To achieve a highly abstracted structural mapping, topics concerning disciplinary distress and social devaluation were contextualized within the mental health dimension. At the same time, physical exhaustion and injury vulnerability were mapped to the physical health dimension, and health-protection deficit was categorized under the social protection dimension.

**Figure 6:**
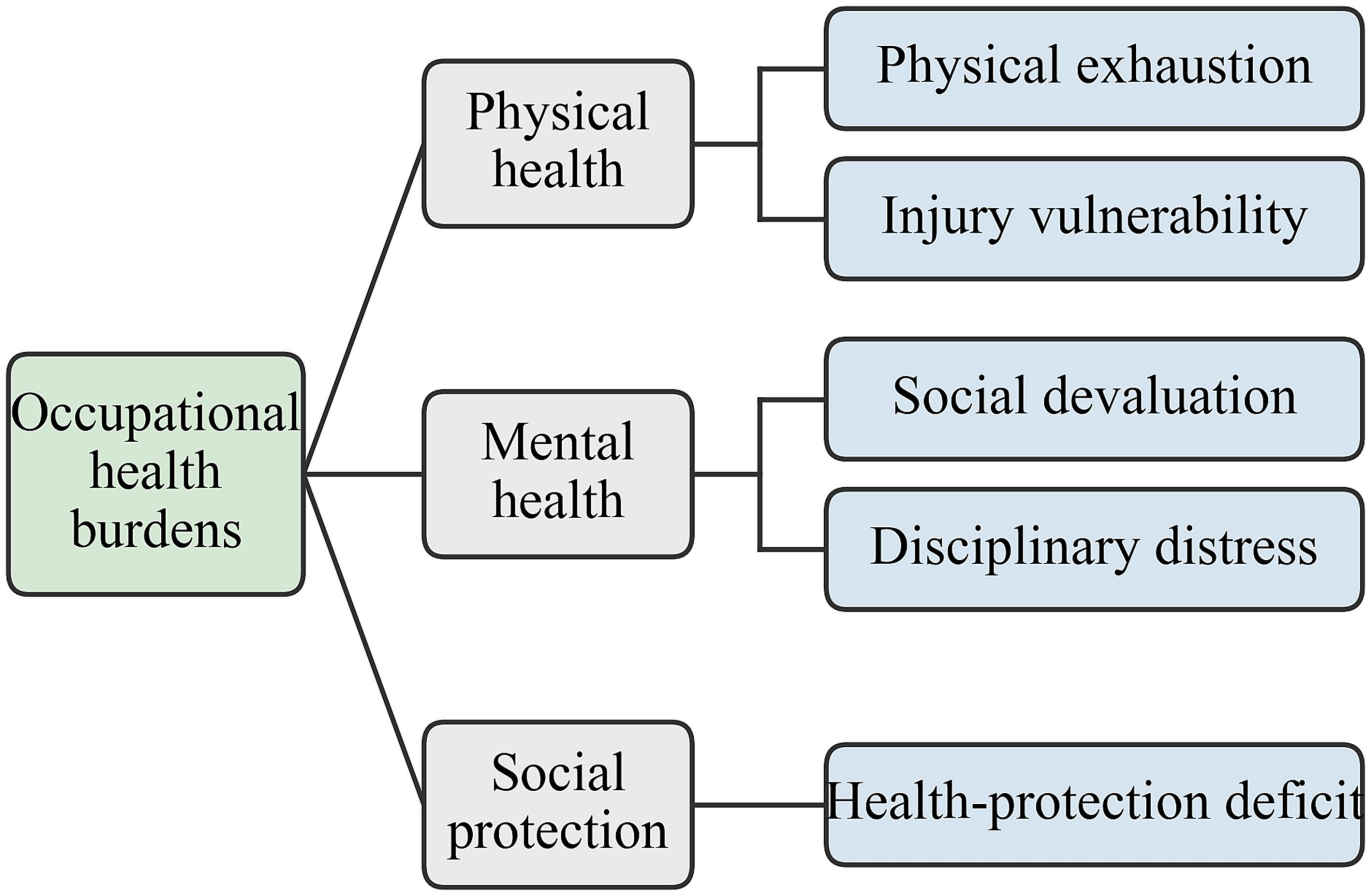
Occupational health burdens under algorithmic management.

Following this structural alignment, topic labels were refined for academic consistency through expert cross-verification (52). This step ensured that standardized terminology accurately reflected these overarching health burden topics. To quantitatively validate the semantic boundaries of these dimensions, a cosine similarity test was conducted. All inter-topic similarity scores remained below 0.05, which confirmed a high degree of distinctiveness and satisfied the precision requirements of the LDA model. These similarity scores, well below the conventional threshold of 0.30, confirm that the five topics capture semantically distinct dimensions of occupational health discourse. Although topics are structurally interconnected through the co-occurrence network, their lexical and semantic cores remain well-separated. The finalized multi-dimensional framework of occupational health burdens under algorithmic management is illustrated in Figure 6.

### 4.2 Topic sentiment intensity analysis

Based on the finalized multi-dimensional framework, a data-driven approach was employed to evaluate the objective prominence and emotional impact of each health burden topic. This text mining approach extracts topic weight and emotional intensity directly from the OFD rider comment corpus. Topic weight reflects the structural probability of each health burden topic across the corpus.

#### 4.2.1 Topic sentiment distribution

The sentiment of each comment was measured using TextBlob (53), a widely used lexicon-based tool for sentiment analysis. It relies on a well-established sentiment lexicon, is openly available, and yields fully reproducible results, properties well suited to the analysis of large volumes of short text. The scoring was applied to the English translations of the rider comments. For each comment, TextBlob returns a polarity score ranging from −1 for the most negative tone to 1 for the most positive tone. Each score was then linearly rescaled to a 0 to 100 range by multiplying it by 50 and adding 50, so that the most negative, the neutral, and the most positive values correspond to 0, 50, and 100. On this scale, scores from 0 to 40 were classified as negative, those from 40 to 60 as neutral, and those from 60 to 100 as positive. The neutral band was centered on the midpoint because scores near 50 correspond to polarity values close to zero, which usually reflect factual statements with little emotional weight. On the original polarity scale, these three bands correspond to polarity values of *p* < −0.2, −0.2 ≤ *p* ≤ 0.2, and *p* > 0.2, respectively.

To capture the affective characteristics of OFD riders’ health-related discourse, the baseline sentiment polarity of the corpus was initially assessed. The data reveal a fundamental characteristic of rider discourse. Neutral sentiment predominates, ranging from 71.57 to 77.16% across all topics. This distribution indicates that the majority of discussions reflect objective accounts of daily operational realities and health-related experiences. Such a neutrality-dominant pattern is consistent with the pragmatic communication style prevalent on short-video platforms, where OFD riders typically describe working conditions and health concerns in factual terms.

**Figure 7:**
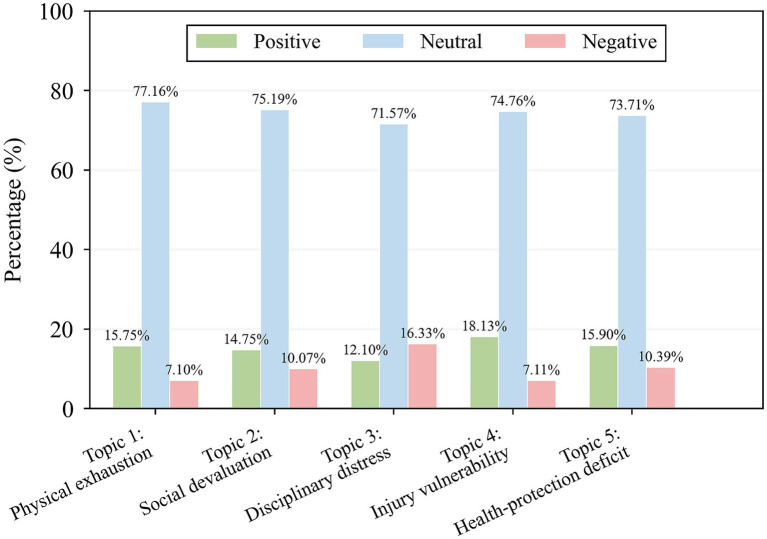
Sentiment polarity distribution.

Critical differences emerge across specific health burden dimensions. Disciplinary distress is the only topic where the negative proportion (16.33%) exceeds the positive proportion (12.10%). This result identifies punitive disciplinary pressure as the primary trigger for rider dissatisfaction and psychological distress. In contrast, physical exhaustion exhibits the lowest negative proportion (7.10%). The distribution of sentiment polarity across the five core health burden topics is illustrated in Figure 7.

Absolute sentiment intensity scores on a 0-to-100 scale were subsequently calculated based on this polarity analysis using the weighted average approach specified in Equation 3. Physical exhaustion yields the highest score of 73.11, which falls within the favorable spectrum. This relatively high sentiment likely reflects the tendency of OFD riders to normalize time pressure as an inherent occupational demand, since they equate delivery speed directly with operational efficiency and income generation. Riders accordingly view time pressure as a routine challenge to be managed. They do not treat it as a health threat to be mitigated.

**Figure 8:**
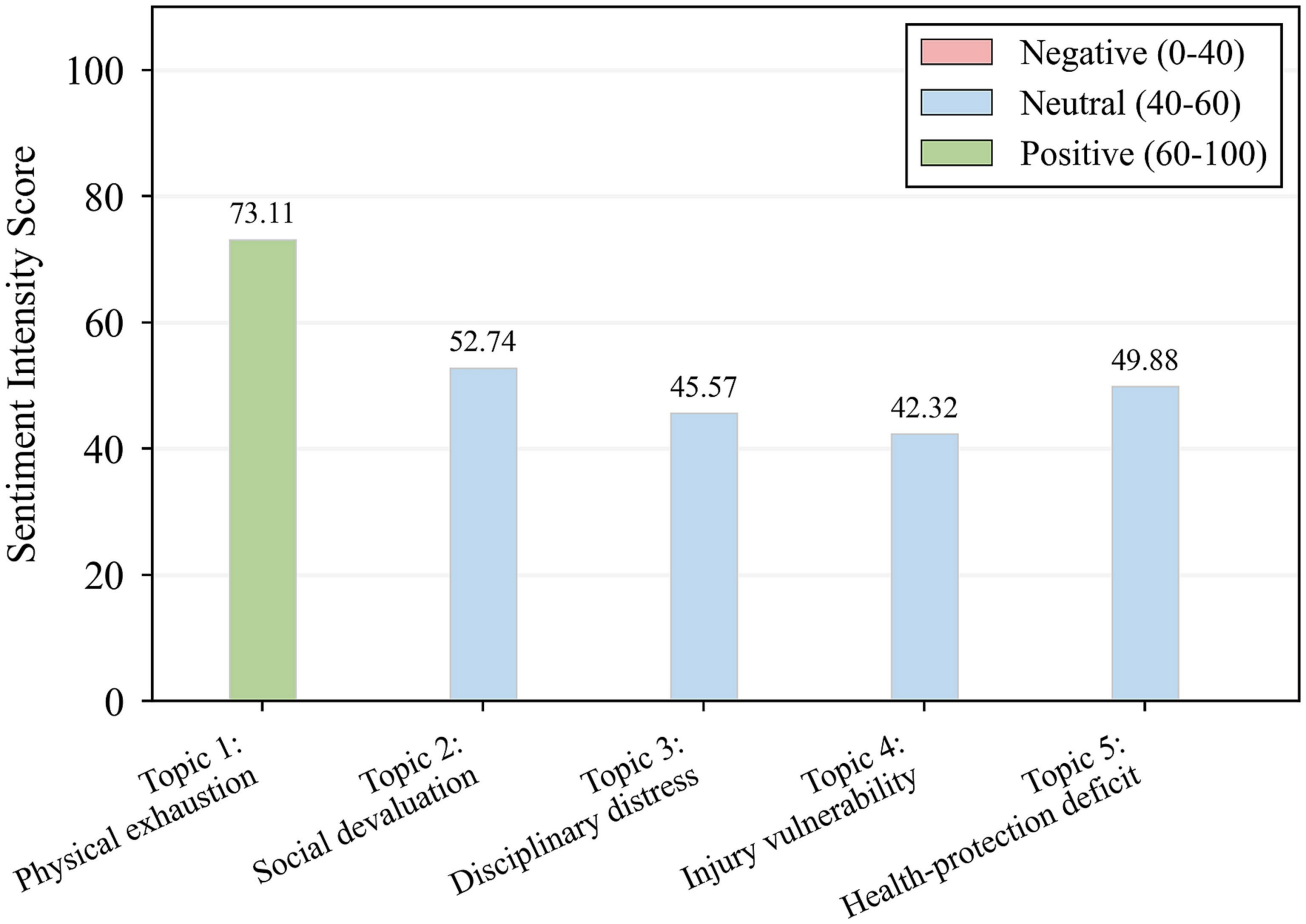
Topic sentiment intensity.

In contrast, injury vulnerability records the lowest score of 42.32, with disciplinary distress close behind, and both fall securely within the unfavorable spectrum. This pattern confirms that the bodily risks of unsafe riding and the punitive algorithmic measures that drive them consistently generate the most substantial psychological resistance and emotional distress despite the moderate overall emotional tone of the corpus. The quantitative spread from 73.11 to 42.32 defines the affective boundaries of the occupational health burden landscape. Detailed sentiment intensity scores by health burden topic are visualized in Figure 8.

#### 4.2.2 Systemic importance and sentiment comparative analysis

Quantifying the systemic impact of each health burden dimension requires evaluating the topics across three dimensions. This evaluation integrates sentiment intensity with degree centrality for structural prominence and betweenness centrality for bridging capability. Specific numerical distributions for the five core health burden topics are detailed in Table 4.

**Table 4:** Normalized centrality and sentiment intensity.

Dilemma dimension	Specific topic	Degree centrality	Betweenness centrality	Normalized sentiment intensity
Physical health	Physical exhaustion	0.2558	0.0799	0.0969
Mental health	Social devaluation	0.2326	0.0177	0.0339
Physical health	Injury vulnerability	0.2302	0.0339	−0.0969
Mental health	Disciplinary distress	0.2093	0.0000	−0.0976
Social protection	Health-protection deficit	0.2093	0.0000	−0.0976

An evaluation of the metrics presented in Table 4 reveals a distinct comparative pattern between structural prominence and emotional impact. Physical exhaustion demonstrates the highest degree centrality of 0.26 and betweenness centrality of 0.08, which exceeds all other topics on both measures. Time compression is thus identified as the most universally discussed occupational health concern and as the central structural hub connecting all other health burden dimensions. Its elevated betweenness centrality further indicates that physical exhaustion functions as the principal conduit through which health burdens in one dimension propagate to others within the network.

While operational pressures dominate the network core, systemic constraints induce the most severe psychological distress. Both disciplinary distress and health-protection deficit display the most extreme negative sentiment intensities, followed closely by injury vulnerability. Such clustering at virtually identical negative extremes suggests that the psychological and safety-related dimensions of occupational health burden form a coherent band of emotional resistance that is qualitatively distinct from the more normalized physical strain.

The betweenness centrality of disciplinary distress equals zero, which highlights a structural paradox within the network topology. A zero betweenness value indicates that platform punishments operate as relatively isolated terminal algorithmic endpoints. They receive pressure transmitted from other nodes without relaying it further. Despite such structural isolation, these endpoint mechanisms elicit the most severe negative psychological responses from OFD riders. The disjunction between structural isolation and emotional intensity suggests that the psychological burden imposed by punitive algorithms is disproportionate to their topological position. Disciplinary mechanisms accordingly function as concentrated points of friction where accumulated systemic pressures converge into acute psychological distress.

**Figure 9:**
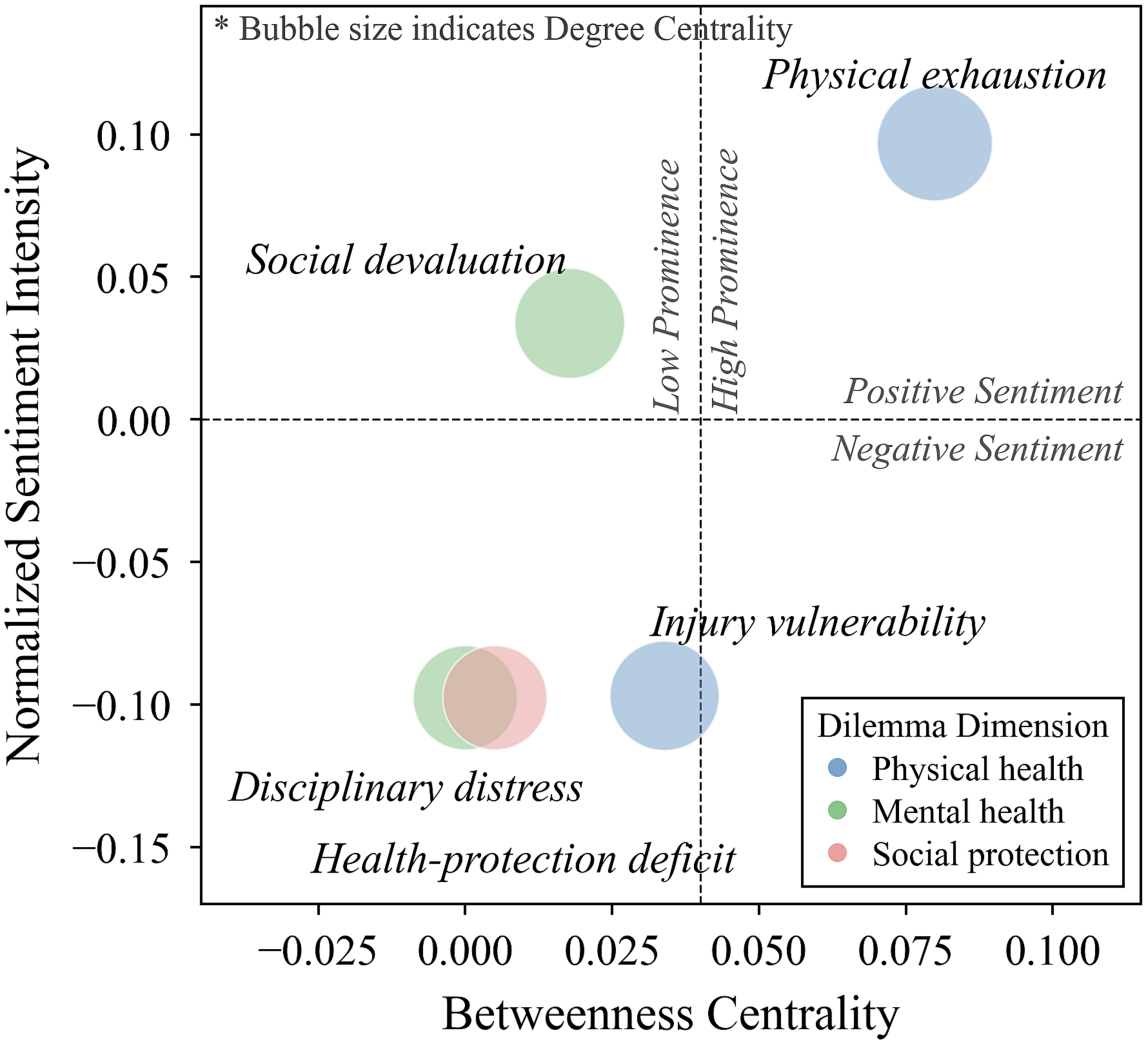
Structural prominence and sentiment intensity.

Sentiment intensity distributions further indicate that disciplinary distress, health-protection deficit, and injury vulnerability constitute the primary drivers of collective negative affective responses. These three topics converge at the negative extreme of the sentiment spectrum, which points to a clear emotional fault line between normalized physical strain and acute psychological and safety-related grievances. When network metrics and sentiment scores are integrated, a structural contrast emerges. Platforms target operational efficiency through time compression, yet the ensuing systemic and punitive burdens generate the most intense health-related friction. This analytical framework grounds the subjective health concerns of OFD riders in objective statistical evidence and confirms that the perceived health burden of algorithmic management is structurally embedded within platform design. To further synthesize these patterns, the two-dimensional quadrant chart presented in Figure 9 visually encapsulates the structural paradox between objective topological prominence and subjective psychological distress.

### 4.3 Topic-related network analysis

As the preceding analysis demonstrates, the identified health burden dimensions are interconnected. To map their structural interactions, a topic co-occurrence network was constructed based on the text mining results. This section evaluates the global topology and node centrality of this network to examine how algorithmic management exacerbates the occupational health burdens experienced by OFD riders.

#### 4.3.1 Global network topology

The co-occurrence network was constructed at the keyword level. For each of the five health burden topics, the 10 keywords with the highest TF-IDF weights were retained, and after merging keywords shared by more than one topic, 44 unique nodes remained. Two keywords were joined by an undirected edge whenever they appeared together in the same rider comment, so the binary adjacency matrix used a co-occurrence threshold of 1 and allowed links across different topics. This procedure produced a network of 44 nodes and 216 edges, whose global properties are summarized in Table 5. At the global level, the network has a density of 0.228, an average degree of 9.818, an average clustering coefficient of 0.948, and a transitivity of 0.788. The density is highly sensitive to network size. A compact network of 44 curated keywords tends to show higher density than one drawn from thousands of terms (54), so density is not directly comparable across networks of different scales. Clustering coefficient and transitivity offer a sounder basis, since both capture local connectivity and stay relatively stable as size changes (55).

**Table 5:** Global topological properties of the network.

Metric	Value
Nodes	44
Edges	216
Network density	0.228
Average degree	9.818
Average clustering coefficient	0.948
Transitivity	0.788

Against published benchmarks in Table 6, the present values are strikingly high. Word co-occurrence networks usually report clustering coefficients of about 0.60 to 0.79, and social networks rarely exceed a transitivity of 0.5 (56, 57), yet the present network surpasses both ranges by a wide margin. The 44 keywords come from five distinct health burden dimensions, so this high triadic closure means that keywords from different dimensions repeatedly appear in the same comments. Riders therefore tend to describe several burdens together within a single account, and terms tied to physical exhaustion often co-occur with those in the injury vulnerability cluster. This interconnection is the most distinctive feature of the network, and it gives network-level evidence that the health burdens of OFD riders are experienced as connected and cascade across dimensions.

**Table 6:** Comparison of network topology metrics with social and co-occurrence networks.

Network (study)	Nodes	Clustering coefficient (C)	Transitivity (T)
This study	44	0.948	0.788
Social networks	$10^{2}-10^{6}$	0.10–0.80	0.05–0.53
Co-occurrence networks	$10^{3}-10^{4}$	0.60–0.79	0.10–0.50

#### 4.3.2 Systemic centrality analysis

Node centrality metrics were evaluated to identify the primary drivers within the algorithmic system. Analysis shows that the platform entity associated with the keyword Meituan demonstrates the highest node-level centrality. Such structural prominence indicates that the platform acts as the primary source of the health burdens observed across all dimensions. The identified health challenges are therefore systemic consequences of centralized algorithmic architecture. They are not isolated individual vulnerabilities.

Secondary core nodes bridge distinct topic clusters and encompass fundamental delivery actions and the customer interface. These nodes represent critical intersections within the network where algorithmic directives and physical delivery tasks interact. A bridging function at these nodes suggests that the customer-rider interface serves as a key transmission point where platform-level algorithmic pressure is converted into individual-level health strain. Targeted regulation of algorithmic constraints at these central nodes could therefore effectively mitigate cascading negative health effects throughout the overall system.

### 4.4 Interview corroboration of the five health-burden dimensions

The thematic analysis of the 32 interviews closely corroborates the five health-burden dimensions extracted from the text corpus, and it further clarifies the mechanisms behind two patterns that the computational analysis could only describe statistically. First, riders’ accounts explain why physical exhaustion, despite its structural centrality, carries a comparatively favorable sentiment score: a subset of participants reframed physical strain as a sign of diligence or fitness rather than as a health threat, which is consistent with the normalization of time pressure reported in Section 4.2. Second, the accounts explain why injury vulnerability and disciplinary distress sit at the negative extreme of the sentiment spectrum: participants described unsafe riding, penalties, and the absence of social protection as arbitrary and largely beyond their control. The correspondence between each interview theme and its health-burden dimension, together with representative excerpts, is summarized in Table 7.

**Table 7:** Interview themes by health-burden dimension.

Health-burden dimension	Interview theme	Corroborating pattern across participants	Representative excerpt
Physical exhaustion	Time pressure and bodily strain	Exhaustion attributed to a timing structure that does not separate cooking from delivery; Chongqing’s slopes and elevator-less buildings intensified the toll.	“The system often dispatches an order before the restaurant has even started cooking, and the countdown begins right away … the time actually left for delivery may be only a few minutes.” (P01)
Disciplinary distress	Fines, ratings and appeals	Penalties and negative reviews were the chief frustration; most saw appeals as ineffective, though a minority reported occasional success.	“I have grown numb to fines … appeals feel like a formality that no one listens to.” (P13)
Injury vulnerability	Traffic risk and accidents	Red-light running and wrong-way riding framed as forced responses to the countdown more than to carelessness; accidents typically self-handled.	“It is not a matter of poor character — the dispatch logic compresses time to the extreme and forces it; I once fell after running a red light and just had to get up and keep delivering.” (P09)
Health-protection deficit	Social and medical insurance	All participants reported only a daily-deducted commercial accident policy; several had self-paid rural medical insurance in their hometowns.	“Proper social insurance is really out of reach for us … the cover arranged through the station feels like it mainly protects the company …, and the platform takes little direct responsibility” (P11)
Social devaluation	Recognition and dignity	Many felt looked down upon and hid their occupation from relatives; a minority, often part-time or younger riders, expressed occupational pride.	“People think we deliver food because we have few other options … I would never let relatives or friends know I do this.” (P17)

Summarizing the coded accounts across the full sample reinforces these patterns while also revealing meaningful heterogeneity. Twenty-nine of the 32 participants described physical exhaustion as a tangible health burden, such as gastritis, lumbar or knee damage, or chronic urinary retention, whereas three (P04, P10, P12) reframed the strain as fitness or personal growth. On disciplinary distress, 29 regarded the appeal process as ineffective or a mere formality, while the same three reported occasionally successful appeals. Twenty-seven participants framed traffic violations as forced responses to the countdown, and five (P04, P10, P12, P30, P32) reported deliberately refusing to violate, two of them after a caution from traffic police. All 32 reported having no employer-provided social insurance and only a daily-deducted commercial accident policy, with five (P03, P08, P12, P20, P32) self-funding rural medical insurance in their hometowns. Finally, 23 described feeling looked down upon and often concealing their occupation, whereas nine (e.g., P04, P10, P12, P25, P30) voiced occupational pride or recounted episodes of customer recognition.

Although most participants worked for Meituan, accounts from riders on Taobao Shangou (formerly Ele.me), and JD Miaosong did not differ substantially in the health burdens described, which suggests that the patterns identified through the single-platform corpus are not unique to one platform. At the same time, a small number of participants, particularly part-time and younger riders, described more positive experiences of autonomy, customer goodwill, and successful grievance resolution, indicating that the burdens documented here, while pervasive, are not uniform across the workforce.

## 5 Discussion

Drawing on the 10,103 preprocessed comments, this section interprets the occupational health challenges among OFD riders through the lens of the topic modeling and network analysis results. Five distinct health burden dimensions have been identified, and the discourse patterns and emotional characteristics embedded in OFD riders’ online comments have been examined. By integrating keyword weights, topological metrics, and sentiment intensities, the following sections contextualize these findings within the theoretical landscape of occupational health under algorithmic management in digital labor platforms.

### 5.1 The occupational health impact of algorithmic efficiency

A central tension within the platform economy is that the efficiency gains produced by algorithmic management come at a significant cost to worker health. By evaluating the central position and emotional distribution of the identified topics within the co-occurrence network, the analysis demonstrates how the pursuit of operational efficiency reshapes the health burden profile of OFD riders. This impact extends beyond direct physical hazards and also affects riders’ psychological well-being, social identity, and access to healthcare.

#### 5.1.1 Efficiency-driven health burden dimensions

The algorithmic system in the platform economy is much more than a dispatch tool. It functions as a comprehensive rule system. This system defines the behavioral boundaries of workers and, by extension, shapes their exposure to occupational health burdens. Among the five identified health burden dimensions, social devaluation occupies a central position in the network, with a degree centrality of 0.2326 that positions it as the second most prominent topic. Such centrality indicates that the platform’s efficiency-first algorithmic logic engenders not only physical but also mental health burdens by systematically devaluing the social status of delivery work. High-frequency terms such as exploitation (0.035), lower class (0.016), and capitalist (0.013) within this topic show that OFD riders perceive algorithmic control as a manifestation of broader structural inequality (58). This perception constitutes a form of occupational stigmatization, which the public health literature recognizes as a risk factor for psychological distress, reduced self-esteem, and diminished help-seeking behavior (59). Riders voiced this stigmatization directly: one described feeling that customers “just treat you as a delivery tool,” with “no recognition at all” (P16). Yet the interviews also revealed a counter-current of occupational dignity. A crowdsourced rider rejected the bottom-of-society label, insisting that “we save money with our own strength, honestly,” and that customers were “actually quite tolerant” (P12); another recalled a customer who messaged to thank her and left a small tip, which made her “heart feel warm” (P02). These divergent accounts indicate that occupational stigma, though widespread, is partially buffered by everyday interpersonal recognition.

In operational terms, physical exhaustion constitutes the most pervasive daily health burden for OFD riders, and the keyword data reflect this pressure directly. Terms such as rider, delivery time, and food preparation form the core vocabulary of this dimension, with characteristic weights of 0.108, 0.035, and 0.021. Among them, rider (0.108) stands as the highest-weighted term across all topics. This weighting highlights the centrality of the individual worker in bearing performance demands and the physical strain they create. Significant disparities in information transparency and bargaining power between the platform and OFD riders exclude riders from the formulation of time estimation rules. Riders lack effective channels to challenge unreasonable algorithmic commands, and real-world uncertainties such as traffic congestion and adverse weather make preset efficiency goals frequently unattainable. Rigid algorithmic rules consequently create acute physical and temporal strain, and they illustrate how platforms optimize order allocation while disproportionately shifting the resulting health burdens onto individual workers. The interview accounts make this temporal structure concrete. One dedicated rider explained that platforms use a “lump-sum total-duration” model in which a nominal 25-min order must absorb the restaurant’s cooking time, waiting, stair-climbing, and traffic alike, so that “once waiting for the food takes too long, the remaining delivery time is simply not enough” (P05). Notably, several riders treated this strain as an ordinary part of the job. One newer rider said he had come to “treat it as doing squats at the gym,” adding that “my body has actually gotten stronger” (P04). This normalization helps explain the comparatively favorable sentiment score that physical exhaustion received in Section 4.2.

#### 5.1.2 Emotional and mental health burden

Long working hours are an established risk factor for cardiovascular disease, stroke, and burnout at the population level (16). As physical and temporal pressures continue to accumulate, OFD riders show significant negative psychological responses. These responses concentrate on health-protection deficit and on the psychological distress induced by occupational stigmatization. The sentiment analysis in Section 4.2 confirms this pattern. As Figure 8 shows, both disciplinary distress and the health-protection deficit fall within the unfavorable sentiment spectrum and remain well below the highest-scoring topic. High-frequency keyword weights corroborate this observation. Exploitation and capital occupy significant proportions of 0.035 and 0.036, respectively, and reflect riders’ critical perception of labor relations and their attendant psychological toll. The frequent appearance of lower class (0.016) suggests that OFD riders possess a clear awareness of their professional marginalization and income precariousness, which contributes to chronic psychosocial stress.

These negative emotional responses stem from a deeper shift in employment models that directly affects worker health. A semantic examination of typical comments under this topic shows that OFD riders are acutely aware of the platform’s tendency to use cooperation models as a means of evading traditional employer responsibilities, including the provision of occupational health protections. Such awareness is reflected in the keyword structure, where compression (0.049), insurance (0.038), and accidents (0.020) collectively form a semantic field centered on health burden exposure and protection deficits. Riders’ anxiety becomes particularly pronounced in high-risk scenarios such as traffic accidents. In these contexts, the keyword insurance reaches a characteristic weight of 0.038. This elevated weight suggests that health-related concerns intensify at the moments when the gap between risk exposure and institutional health protection is most acutely felt. Interviewees articulated this awareness in plain terms. One observed that because riders “count as flexible employment, the platform does not have to bear the responsibilities of formal employment” (P24). Several reported that their only coverage was a daily-deducted commercial accident policy, and that any medical insurance had been arranged and paid for by themselves in their rural hometowns, with the platform bearing “no responsibility at all” (P03). These accounts ground the health-protection deficit dimension in concrete institutional gaps that go beyond discourse.

The formation of health-related anxiety among OFD riders is intrinsically linked to platform operational strategy. Platform enterprises predominantly adopt a light-asset model and focus on algorithmic iteration and data management. These enterprises concurrently transfer the physical and financial risks of labor reproduction to individual riders through technical contractual arrangements (60). Transferred costs include vehicle maintenance, personal accident risks, and medical expenses. Such a transfer stands in sharp contrast to the job autonomy heavily promoted during the early stages of the platform economy. When riders face strict digital monitoring and at the same time lack basic health protections, their sense of health vulnerability turns into clear dissatisfaction. These findings confirm a paradox in algorithmically managed labor processes. Technological advancements improve overall productivity but often exacerbate the health vulnerability of workers. This outcome arises because algorithmic management restructures traditional contractual relationships and displaces employer health protection obligations onto individual workers, which creates what the occupational health literature describes as precarious employment conditions associated with elevated disease risk and reduced access to healthcare (14).

### 5.2 Cascading connections among health burden dimensions

As the network analysis makes clear, these health burdens do not exist in isolation. They interact within the platform’s algorithmic control system and cascade into a compounding web. Worker health vulnerabilities therefore grow out of the interplay between entangled management practices and operational constraints. The structural topology of the co-occurrence network provides the foundation for examining how efficiency-driven pressures transmit across health burden dimensions.

#### 5.2.1 Cascading transmission of health burdens

**Figure 10:**
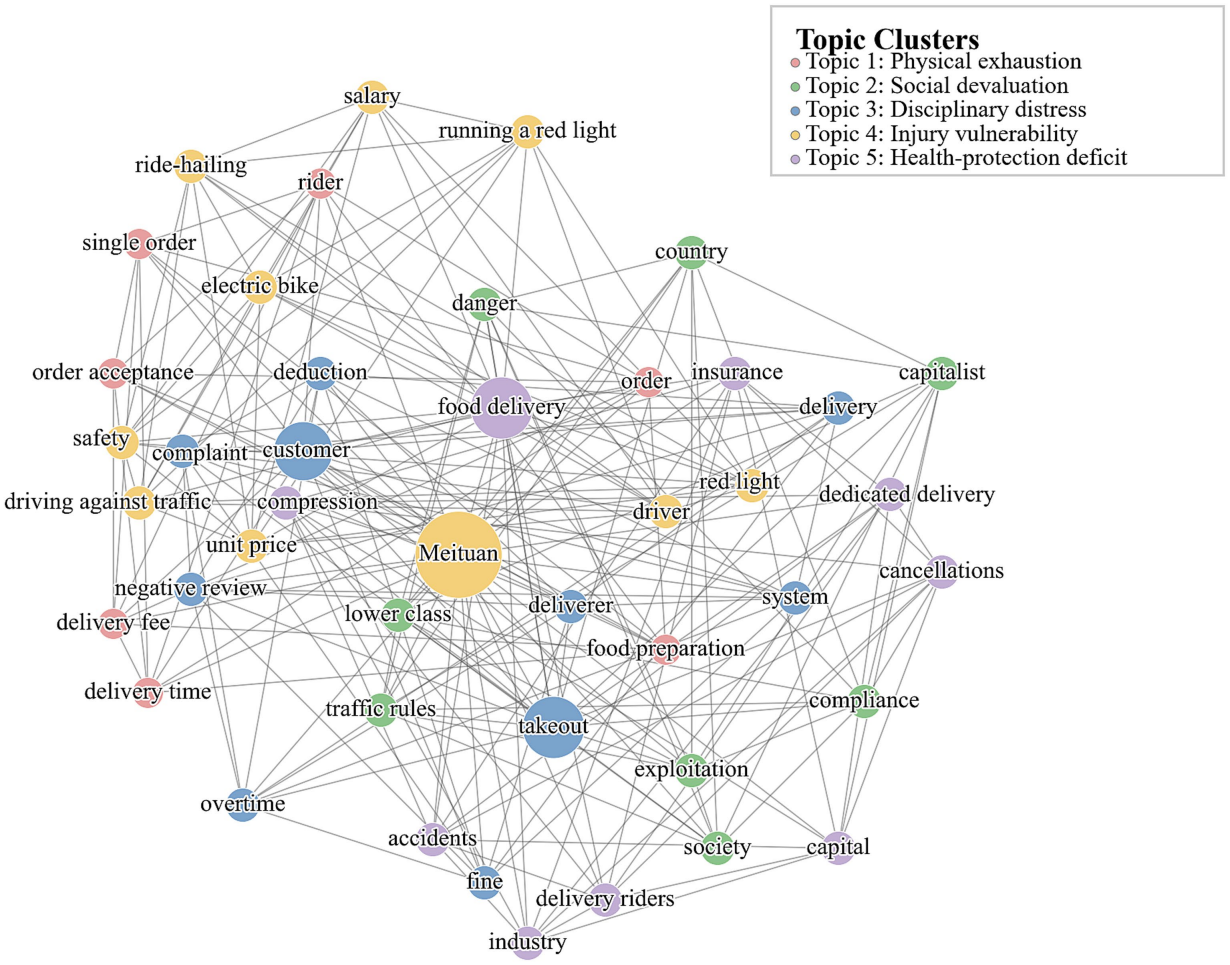
Global co-occurrence network.

The multi-dimensional health burdens outlined above function as an interconnected whole, bound together by complex management practices. Table 5 presents the global metrics of the topic co-occurrence network. The high clustering coefficient and transitivity values confirm structural cohesion and point to robust local connectivity. Any adjustment of algorithmic strategies within one dimension will therefore trigger chain reactions across the entire health burden network. To illustrate these compounding pathways, the complete topological structure of this network is mapped in Figure 10.

Within this topological structure, health burden transmission follows a pattern of unidirectional constraint and hierarchical progression. Pressure is transmitted downward through punitive disciplinary mechanisms. Terms denoting direct economic penalty attributes include overtime, negative review, deduction, and fine. These terms are intimately correlated with delivery efficiency requirements. Their characteristic weights are 0.107, 0.024, 0.020, and 0.017, respectively. This correlation pattern reveals a hierarchical health burden architecture. The platform first establishes time-based performance benchmarks through its algorithmic system. Compliance is then enforced through an automated penalty mechanism that converts delivery anomalies into direct income deductions. This dual-layered structure secures the platform’s dominant position in work management and simultaneously amplifies the mental health burden on riders. When delays or anomalies in terminal delivery are detected by the algorithmic system, rider income is automatically adjusted through penalty mechanisms, which transforms invisible algorithmic logic into tangible economic and psychological consequences. Riders’ descriptions of the penalty mechanism underscore its perceived arbitrariness. One recounted being docked for being “a minute late,” with malicious negative reviews deducted “without even verifying” and appeals leading nowhere (P14). This sense of futility was not universal, however: one part-time rider described a service agent who “checked the road conditions carefully” and refunded an unfair penalty, concluding that “if you are in the right, you can sometimes get through” (P10). Such counter-cases suggest that the distress attached to disciplinary mechanisms stems less from the penalties themselves than from the opacity and inconsistency of the appeal process.

Such unidirectional pressure transmission creates a significant health-relevant power imbalance between the platform and OFD riders. Riders bear both the economic and health consequences of algorithmic decisions but lack effective channels to review or negotiate these rules. Addressing this imbalance requires external intervention from public health and labor regulatory bodies.

Government agencies should establish binding algorithmic transparency mechanisms to reduce information asymmetry and to ensure that management rules account for occupational health considerations. Regulations could require platforms to disclose their core algorithmic parameters to both riders and regulators. These parameters include time estimation, dispatch weights, distance accounting, and reward-penalty thresholds.

At the same time, platforms should optimize channels for rider input and establish consultation mechanisms with rider representatives. Such an inclusive approach would ensure that reasonable demands regarding labor intensity, rest periods, and delivery times are accurately reflected in parameter settings. Such reflection would reduce the excessive job demands that the JD-R model identifies as primary drivers of health impairment (20). When invisible algorithmic logic materializes as visible income reduction, compressing delivery time becomes the only variable that OFD riders can autonomously regulate. Riders then rely on increased physical exertion to shorten delivery time and maintain their livelihood. This compensatory behavior further elevates physical health burdens through fatigue accumulation.

#### 5.2.2 Compounding health effects across dimensions

The cascading transmission of efficiency pressures inevitably forces OFD riders into a severe conflict between delivery timeliness and traffic safety, with direct consequences for injury vulnerability. The current operational model is dominated by efficiency goals and reinforced by rigid appraisal mechanisms, and indirectly but significantly increases the probability of traffic-related injuries. Within the injury vulnerability topic, running a red light and driving against traffic occupy central network positions with high weights of 0.134 and 0.042, respectively. Running a red light, with a weight of 0.134, represents one of the highest individual keyword weights across the entire corpus. This finding highlights its centrality in rider discourse and its significance as an occupational safety concern.

Further contextual analysis indicates that high-frequency traffic violations extend beyond a simple lack of individual safety awareness. Within the corpus, OFD riders frequently justify these behaviors as pragmatic responses to algorithmic constraints, and many explicitly state that violations would cease given adequate delivery time. Such accounts suggest a direct connection between platform operating rules and unsafe riding behavior that elevates injury vulnerability. The keyword unit price carries a substantial weight of 0.033, which highlights a severe survival-versus-safety trade-off. Compressed per-order compensation forces riders to accept more orders to maintain a living wage. Rigid algorithmic time limits make compliant driving nearly impossible under such high delivery volume. Traffic violations consequently become a calculated strategy to satisfy appraisal requirements and secure a basic income. This dynamic illustrates a systemic compounding effect in which algorithmic rigidity and economic pressure jointly erode occupational safety standards and elevate the population-level burden of traffic-related injuries. The interviews echo this reasoning. One rider framed traffic violations as a survival calculation, explaining that “the pressure of overtime deductions hangs overhead,” forcing riders to “break the rules to trade for time” (P21). Crucially, when that pressure was removed, behavior changed: another participant reported that after being cautioned by a traffic officer he would “rather work for nothing than break the rules,” because “my life is my own” (P32). This contrast reinforces the interpretation that unsafe riding is structurally produced, and it points to algorithmic time parameters over rider education as the effective lever for injury prevention.

**Figure 11:**
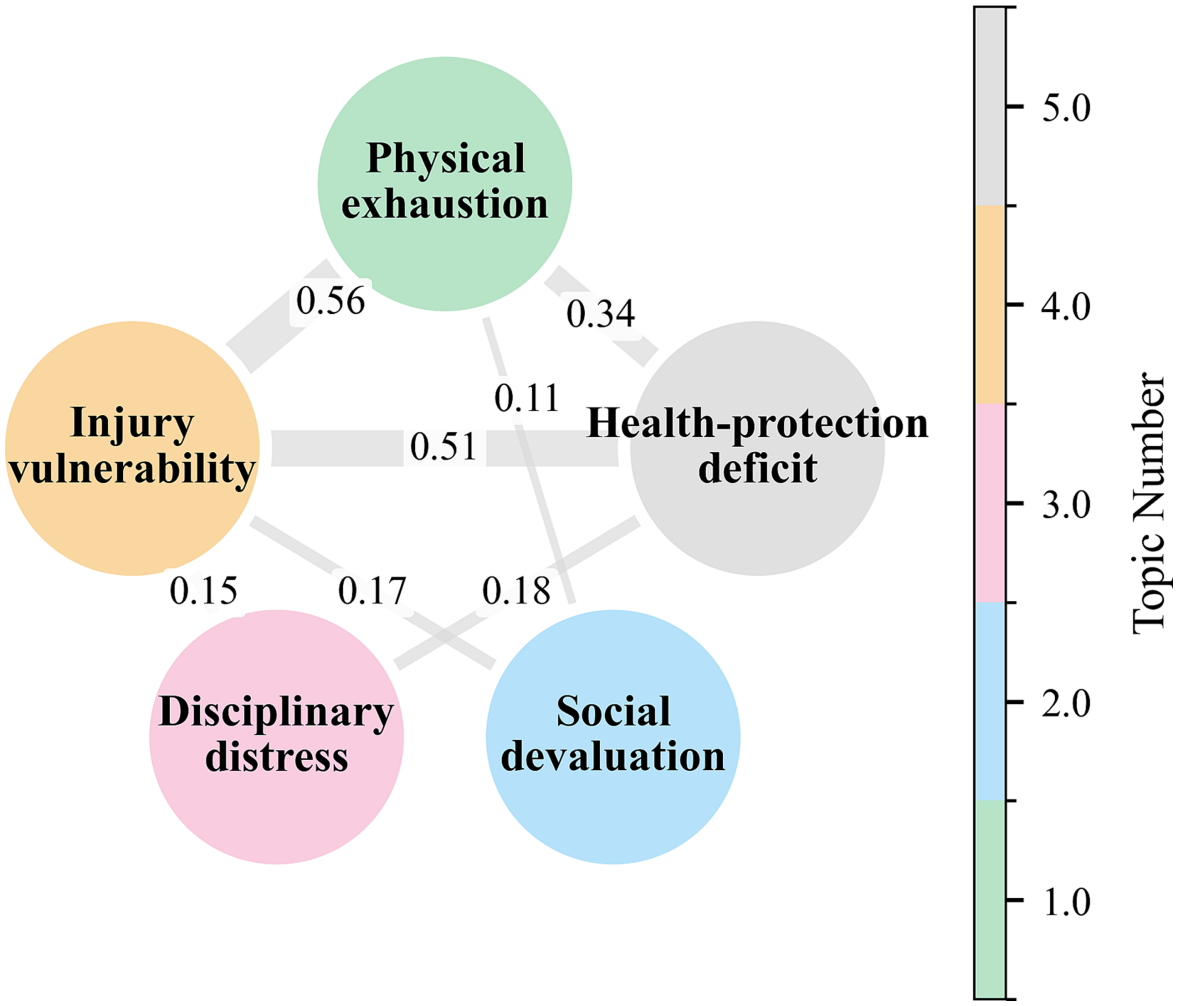
Core correlation network.

The co-occurrence network substantiates this compounding mechanism. Direct cross-cluster linkages between physical exhaustion and injury vulnerability keywords confirm that time compression is structurally conducive to unsafe riding behaviors. The core correlation network presented in Figure 11 further illustrates how these high-centrality health burden nodes interconnect. The connection between physical exhaustion and injury vulnerability carries a high edge weight of 0.56, which demonstrates that platform-level time pressure directly translates into elevated injury risk. From a public health perspective, this finding indicates that occupational injury prevention among OFD riders cannot be achieved through individual-level safety education alone. Road traffic injury remains one of the leading causes of work-related death globally, and international public health agencies have identified platform-based delivery work as an emerging occupational safety concern (12). It requires upstream intervention targeting the algorithmic parameters that generate time pressure.

Addressing this structural health challenge requires a shift from unilateral administrative control toward governing the platform’s core algorithmic logic with health protection as a design principle. Governments should enhance labor protection regulations to promote more equitable compensation structures, which would directly reduce the income pressure that incentivizes unsafe riding. Platforms should embed elastic adjustment and fault-tolerance mechanisms into their appraisal systems. Practical measures that would mitigate the unidirectional transfer of health burden from platform to rider include automatic deadline extensions during extreme weather conditions, real-time anomaly reporting for uncontrollable delays, and graduated penalty thresholds that prevent single incidents from producing disproportionate economic consequences.

In parallel, OFD riders should draw on industry unions and worker associations to enhance collective bargaining capabilities and to establish shared occupational safety norms. A coordinated approach involving government regulation, platform responsibility, and active worker participation is essential to optimize the distribution of health burdens across the platform labor system. Such a collaborative governance framework enables stakeholders to alleviate the detrimental health impact of rigid algorithmic rules and to foster the sustainable and health-protective development of new employment models within the digital economy.

## 6 Conclusion

This study demonstrates how the efficiency pursued by algorithmic management becomes a cascading health burden for OFD riders, and generates multi-dimensional occupational health burdens across physical, psychological, and social protection dimensions. Employing LDA-based semantic mining, structural network analysis, and sentiment intensity assessment as an integrated analytical framework, the findings reveal that the efficiency-first logic of algorithms systematically restructures the occupational health burden profile of OFD riders. Five distinct health burden dimensions were identified and validated through expert review and semantic distinctiveness testing. The co-occurrence network reveals that these dimensions form a tightly interwoven system and do not act as isolated challenges. Physical strain from time pressure cascades through disciplinary mechanisms into injury vulnerability and psychological distress. These findings corroborate the theoretical proposition that algorithmic management functions as a structural determinant of occupational health in the platform economy and establish a foundation for targeted public health interventions.

### 6.1 Contributions

At the theoretical level, this study extends the JD-R model from the interaction of separate demands and resources toward the structural transmission of occupational health burdens. The co-occurrence network shows that algorithmic demands and resource deficits operate together and not in isolation. They form a connected system, and within it pressure cascades from physical strain through disciplinary mechanisms into injury vulnerability and psychological distress. A second theoretical insight concerns a structural paradox. Disciplinary mechanisms hold a peripheral position in the network, yet they trigger the strongest negative responses among riders. This gap between structural position and emotional intensity shows how systemic pressure concentrates into acute distress at the endpoints of the algorithmic system. It also moves the analysis toward the direct interaction between opaque algorithmic mechanisms and worker well-being.

At the methodological level, the study develops an integrated mixed-methods framework for occupational health research in the platform economy. It applies LDA topic modeling, co-occurrence network analysis, and sentiment intensity assessment to large-scale rider discourse, and it adds semi-structured interviews. The computational strand extracts collective health perspectives at the population level. The interviews corroborate the five health burden dimensions and ground them in concrete rider experience. This design captures both breadth and depth, and it offers a transferable template for occupational health research in other algorithmically managed settings.

At the practical level, the findings translate into concrete and coordinated governance measures. The burdens transmit across dimensions, so intervention in upstream algorithmic parameters such as time estimation and penalty thresholds can produce benefits across several dimensions at once. The detailed proposals for government, platforms, rider organizations, and industry associations appear in the following section.

### 6.2 Public health policy implications

The finding that algorithmic efficiency can turn into a health burden carries clear implications for occupational health governance, and the structure of the burdens shows where governance should concentrate. Our analysis shows that the five burdens do not stand apart. Physical exhaustion sits at the center of the network and transmits pressure into injury vulnerability and social protection deficits, while disciplinary distress remains structurally peripheral yet draws the strongest negative sentiment from riders. This gap between structural position and felt intensity exposes a limit in any purely complaint-driven approach. The burden riders voice most loudly is not the one that connects the system, so governance that follows expressed grievance alone would direct effort toward the visible margin and leave the structural core untouched. The measures below therefore read the burden network as a whole, and the action of each party prepares the ground for the next one.

Government is best positioned to regulate a burden on the basis of its structural position in the network. Physical exhaustion anchors the network, but riders treat time pressure as ordinary, so this central node generates little protest and would attract little attention under complaint-driven oversight. Regulators should therefore target the parameters that sit upstream of it—time estimation, dispatch weighting, and the thresholds that convert delays into income loss. Two measures follow directly: platforms should be required to disclose these parameters, and their automated penalty decisions should be opened to independent review. Placing scrutiny at this upstream point addresses not only exhaustion itself but the injury and protection burdens that cascade from it.

Platforms operate the technical system through which the cascade runs, and this gives them a matching responsibility to treat the burdens as a connected set. Two redesigns would carry the principle into practice. First, dispatch logic should be evaluated by its estimated load on rider recovery alongside delivery speed, so that the health cost of a routing decision becomes visible at the moment it is made. Second, the penalty system, which compounds this pressure and produces the disciplinary distress that riders resent most sharply, should record each rider objection and return a reasoned reply. This change would convert one-way punishment into a two-way exchange and make the platform accountable for the burden it concentrates at the network margin.

Rider organizations give individual workers a collective voice, and our results locate where that voice matters most. Disciplinary distress stays peripheral in the network and rarely registers in routine operational metrics, yet it carries the sharpest negative sentiment—precisely the kind of burden that aggregate performance data conceal and that organized representation can bring to light. Their central contribution would be to operate an accessible complaint channel that lets riders report unsafe conditions and contest penalties. The records it generates would give platforms and regulators a direct reading of a burden that standard indicators miss, recovering the part of the health picture those indicators erase.

Industry associations can build shared instruments that no single platform could establish alone, and two deserve priority. The first is social protection: our interviews show that most riders hold only a daily commercial accident policy and carry no employer social insurance, so associations should help design portable mechanisms that attach occupational injury insurance and healthcare to each rider’s cumulative work record and follow the worker across platforms. The second is a cross-platform metric that tracks structural centrality against sentiment for every burden dimension. A widening gap between the two would flag a structurally minor burden turning acute, as disciplinary distress does here, and would convert the analysis of this study into a standing tool for ongoing monitoring.

These measures address the separate burden dimensions and also reach the pathways that connect them. The four parties form one interconnected system. Government scrutiny of upstream parameters sets the conditions for platform redesign, organized representation recovers the burdens that metrics hide, and shared instruments let associations track how the network shifts over time. Because the burdens transmit from one dimension to another, effort spent at an upstream node returns benefits across several dimensions at once, and reading structure alongside sentiment shows which node to choose. A governance approach built on the shape of the burden network offers a more precise response than one that treats each dimension, or each complaint, on its own.

### 6.3 Limitations

This study examines the occupational health burdens of algorithmic management, and several limitations should be acknowledged. First, the analytical framework relies mainly on social media discourse. As Section 3.3 explains, the text-derived topics reflect the thematic structure of rider discourse, and they are not clinically measured health outcomes. The health burden label assigned to each topic is an interpretive inference supported by keyword semantics and expert validation. It is not a direct measurement of physiological or mental health status. The semi-structured interviews partly offset this limitation. Future research would benefit from triangulating these findings with physiological measures of fatigue and stress, validated psychometric instruments, clinical surveys, medical records, and occupational injury registries. Such triangulation would link discursive patterns to objective health outcomes. The study also relied on LDA. Future work could complement it with neural transformer models such as BERTopic and with neural topic models to capture richer contextual semantics.

Second, the study focuses on OFD riders in China, and it draws on a single social media platform within a single national context. Such data capture publicly expressed concerns, but they may miss riders who avoid these platforms or are reluctant to discuss health online. Douyin users also skew younger, more urban, and more digitally active. The findings may therefore underrepresent older riders, riders with lower digital literacy, and riders in less urban areas. These gaps bear directly on health equity within the rider population. The evidence comes from one platform and one country, so cross-platform and cross-national transfer of the findings remains limited. Future research should extend the framework to comparable groups such as ride-hailing drivers and logistics couriers. It should also apply the framework to other countries where labor regulation, healthcare systems, and norms around health disclosure differ.

Third, the design is cross-sectional. The co-occurrence network reveals associations among the health burden dimensions, but it cannot establish their temporal order or causal direction. The cascading pathways described here are therefore interpretive, and they are not confirmed sequences. Longitudinal designs that track rider health before and after specific algorithmic or policy changes would test these pathways directly. They would also strengthen the causal claims that the present design cannot fully support.

## Data Availability

The raw data supporting the conclusions of this article will be made available by the authors, without undue reservation.
